# Segmental Zoster Paresis With Comorbid Carpal Tunnel Syndrome

**DOI:** 10.7759/cureus.97150

**Published:** 2025-11-18

**Authors:** Rachel M Bristow, Charis Won, Nissy Lee, Dwight Scott, Joseph E Brooks

**Affiliations:** 1 Physical Medicine and Rehabilitation, New York Institute of Technology College of Osteopathic Medicine, Jonesboro, USA; 2 Physical Medicine and Rehabilitation, Vitality Physical Medicine, Davenport, USA

**Keywords:** carpal tunnel syndrome, edx, electrodiagnostic studies, electromyography, emg, herpes zoster, segmental zoster paresis, varicella-zoster virus (vzv)

## Abstract

This case report involves a 63-year-old male who presented with acute right deltoid weakness following a shingles outbreak in the C6 dermatome, with a chronic history of carpal tunnel syndrome. Notably, the patient had an overuse injury to his shoulder and received a cortisone injection prior to the shingles outbreak. Electrodiagnostic (EDX) testing initially revealed right C6 acute/subacute cervical radiculitis and right severe median neuropathy at the wrist with significant axonal loss. Repeat EDX studies five months following the onset of shoulder paresis showed improved motor recruitment and reinnervation of the C6 lesion, while the median neuropathy findings remain unchanged. This case highlights the diagnostic complexity of concurrent proximal and distal nerve pathologies, often referred to as double crush syndrome, as well as considerations for management and natural history of segmental zoster paresis.

## Introduction

Segmental zoster paresis is a rare complication of herpes zoster that may mimic cervical radiculopathy. When it occurs in a patient with coexisting neuropathy, diagnostic clarity becomes challenging. We describe a case of segmental zoster paresis in a patient with underlying carpal tunnel syndrome (CTS), illustrating the complexity of overlapping nerve pathologies and the essential role of electrodiagnostic (EDX) studies.

Cervical radiculopathy typically arises from disc herniation, degeneration, or spondylotic changes that compress the nerve root, producing pain radiating to the upper extremity and, at times, weakness and diminished reflexes [1]. In rare cases, inflammatory causes, such as herpes zoster, produce a similar clinical picture. This overlap may result in delayed recognition of segmental zoster paresis without careful electrodiagnostic evaluation.

## Case presentation

A 63-year-old man with a long-standing history of right-sided carpal tunnel syndrome presented with new-onset right deltoid weakness following a shingles outbreak in the C6 dermatome. Several days prior to the outbreak, he received a corticosteroid injection in the right shoulder for presumed musculoskeletal pain related to an overuse injury. In February 2025, he was evaluated one month after the shingles outbreak, which was treated with a course of acyclovir; at which time he reported persistent numbness and weakness since the onset.

Physical examination revealed limited passive cervical side bending to the right, with normal side bending to the left and normal bilateral cervical rotation. Spurling's exam was negative. Strength testing showed 4/5 in right shoulder flexion, elbow flexion, and external rotation, with all other muscle groups 5/5. Sensory testing demonstrated decreased light touch sensation in the C6 dermatome, with normal sensation elsewhere. Deep tendon reflexes were 2+ in the left biceps, triceps, and brachioradialis; 1+ in the right biceps; 2+ in the right triceps; and absent in the right brachioradialis.

Initial EDX studies (February 2025) revealed acute/subacute right C6 cervical radiculitis and severe median neuropathy at the wrist with significant axonal loss (Tables 1, 2).

**Table 1:** Nerve conduction study findings at the wrist; initial findings in February and follow-up in June 2025. ms: milliseconds; mV: microvolts; FU: follow-up study.

Nerve	Latency		Amplitude	
	Initial	FU	Initial	FU
Right median (motor)	7.7 ms	6.9 ms	3.3 mV	3.7 mV
Right ulnar (motor)	3.7 ms	3.8 ms	10.3 mV	10.4 mV

**Table 2:** Electromyography study findings of the right upper extremity; initial findings in February and follow-up in June 2025. N: normal; FU: follow-up study; INC: increased; RED: reduced. Insertional activity was not documented in the table; results were normal for all muscles tested at both the initial and follow-up visits.

Muscle	Polyphasic motor unit potential		Fibrillations		Positive waves		Duration		Recruitment		Inference patterns	
	Initial	FU	Initial	FU	Initial	FU	Initial	FU	Initial	FU	Initial	FU
Deltoid	3+	2+	3+	1+	3+	N	INC	INC	RED	RED	N	N
Biceps	0	1+	N	N	N	N	N	INC	N	RED	N	N
Triceps	0	1+	N	N	N	N	N	INC	N	RED	N	N
Pronator teres	2+	2+	1+	N	N	N	N	N	RED	RED	N	N
Abductor pollicis brevis	0	0	N	N	N	N	N	N	N	RED	N	N
First dorsal interosseous	0	0	N	N	N	N	N	N	N	N	N	N
Brachioradialis	0	3+	N	N	N	N	N	INC	RED	RED	N	N

The patient opted for conservative management with physical therapy. On follow-up surgical evaluation, the patient was not recommended for spine surgery due to the inflammatory nature of the radiculitis. At the four-month follow-up evaluation, he reported gradual improvement in shoulder active range of motion and strength testing. Repeat EDX studies (June 2025) showed significant electrophysiologic evidence of reinnervation (Table 2) with resolving denervation potentials and reduced motor unit recruitment in the right C6 myotome, while the severe median neuropathy remains relatively unchanged, with similar median motor onset latency and amplitude (Table 1). These findings, on needle electromyography (EMG), suggested collateral sprouting with reinnervation.

## Discussion

This case illustrates the diagnostic complexity of overlapping neuropathies. Segmental zoster paresis is an uncommon manifestation of varicella-zoster virus (VZV) reactivation, which occurs when latent virus in the dorsal root ganglia becomes active, often in the context of immune compromise. Segmental zoster paresis has been reported in only 0.5-5% of herpes zoster cases [1]. It presents as focal motor weakness in the affected myotome and can closely mimic degenerative cervical radiculopathy or entrapment neuropathy, particularly when the rash is subtle or precedes weakness [2]. Prognosis is generally favorable, with approximately two-thirds of patients achieving full or near-complete recovery [3]. In this patient, serial EDX studies were critical in clarifying the etiology, documenting improvement, and preventing unnecessary spine surgery.

Despite reinnervation of the C6 myotome, the patient’s severe median neuropathy persisted, underscoring the “double crush” phenomenon in which proximal and distal lesions coexist. Differentiating the relative contribution of each site is essential when formulating a management plan. Standard treatment for severe carpal tunnel syndrome (CTS) is surgical decompression; however, anesthesia choice requires caution in the setting of recent nerve injury. Regional blocks can exacerbate or mask deficits, whereas local anesthesia carries a lower neurologic risk [4,5]. A shared decision-making approach remains ongoing to balance the patient’s interest in surgical CTS treatment against the need to protect the healing cervical nerve.

Broader context of varicella-zoster virus neurologic complications

VZV reactivation produces a wide spectrum of neurologic sequelae. The most frequent is post-herpetic neuralgia, characterized by persistent neuropathic pain beyond resolution of the rash. Motor involvement, as in segmental zoster paresis, represents a rarer but clinically significant complication of peripheral nervous system infection. Mononeuropathies affecting distal nerves, including isolated median or ulnar neuropathies, have been described and may be misdiagnosed as primary entrapment syndromes [6]. Cranial nerve involvement is also well recognized. Ramsay Hunt syndrome (CN VII) causes vesicular otic rash, ear pain, and facial paralysis, while herpes zoster ophthalmicus (CN V1) may produce ocular complications and extraocular muscle palsies [7,8]. Central nervous system complications, such as VZV meningitis, encephalitis, transverse myelitis, and vasculopathy, can lead to stroke, particularly in elderly or immunocompromised patients [9]. This broad spectrum highlights that shingles is not merely a benign cutaneous eruption but a multisystem disease requiring vigilance for delayed motor or central deficits.

Management of carpal tunnel syndrome in the setting of recent varicella-zoster virus

CTS is the most common entrapment neuropathy, affecting 1-5% of the general population, and results from median nerve compression within the carpal tunnel [10,11]. Early symptoms typically include paresthesia and pain in the radial digits, with motor weakness appearing later as demyelination and axonal injury progress. Management options range from splinting and glucocorticoid injection to surgical decompression, depending on severity [10].

Regarding surgical management for patients with recent VZV-related nerve injury, anesthetic technique is especially important. Regional brachial plexus blocks may worsen or obscure pre-existing deficits, whereas local anesthesia is safer and is recommended by the American Academy of Orthopedic Surgeons for carpal tunnel release [4,5]. When local anesthesia is used, lidocaine with or without epinephrine is preferred; longer-acting agents such as bupivacaine may hinder neurologic monitoring in patients with unstable nerve function [12]. Emerging minimally invasive techniques, including ultrasound-guided thread release, perineural hydrodissection, and balloon carpal tunnelplasty, show promise but have not been studied in the context of recent VZV infection [13]. Any procedure that disrupts tissues in a recently affected dermatome carries an elevated risk of neurologic complications and should be carefully weighed against potential benefits [14].

Role of osteopathic manipulative treatment (OMT)

Conservative measures remain valuable for both CTS and cervical radiculopathy during the recovery phase. OMT techniques designed to decompress the median nerve, such as transverse extension, thenar extension, and opponens roll, have been shown to improve hand function and reduce symptoms by mobilizing the transverse carpal ligament and enhancing nerve glide [15]. OMT can also address cervical radiculopathy by targeting somatic dysfunctions of the cervical and thoracic regions using counterstrain, myofascial release, and balanced ligamentous tension [15]. In this case, OMT provided symptomatic relief and an additional non-surgical option while the patient's C6 nerve root continued to recover.

## Conclusions

Sequential EDX studies are pivotal for distinguishing overlapping upper-extremity nerve pathologies, particularly in cases of double crush syndrome complicated by post-herpetic motor involvement. These studies provide objective data to track recovery, guide management, and ensure that interventions, such as surgical timing and anesthesia choice, are tailored to protect vulnerable nerves. This case highlights the importance of recognizing segmental zoster paresis in the context of concurrent neuropathies and underscores the role of conservative management and staged decision-making in optimizing patient outcomes.
